# Targeting synergetic endothelial inflammation by inhibiting NFKB and JAK-STAT pathways

**DOI:** 10.1016/j.isci.2025.113307

**Published:** 2025-08-07

**Authors:** Stijn A. Groten, Pieter Langerhorst, Georgios Malamas, Alastair Barraclough, Arie J. Hoogendijk, Maartje van den Biggelaar

**Affiliations:** 1 Department of Molecular Hematology, Sanquin Research, Plesmanlaan 125, 1066 CX Amsterdam, the Netherlands

**Keywords:** Biochemistry, Cell biology, Proteomics

## Abstract

Multiple systemic vascular inflammatory disorders are associated with endothelial dysfunction and elevated levels of TNFα and IFNγ. Combined TNFα and IFNγ stimulation induces synergetic hyperinflammation in endothelial cells (ECs) through the activation of the NFKB and JAK/STAT pathways. Here, we assess how targeting these pathways affects EC inflammation. Using mass spectrometry based proteomics, we investigate system-wide effects of TNFα- and IFNγ-stimulated Endothelial Colony Forming Cells (ECFCs) in combination with inhibitors targeting NFKB and JAK/STAT pathways. JAK1 inhibitor itacitinib blocked IFNγ-, but not TNFα-induced proteomic responses. IKK2/STAT3 inhibitor TPCA1 attenuated both responses. Most TNFα+IFNγ-induced proteins, such as pyroptosis mediators, chemokines, and Weibel-Palade Body content, were inhibited by both inhibitors, highlighting their synergetic dependency on both pathways. Imaging of Von Willebrand Factor (VWF) revealed an extracellular VWF network induced by combined stimulation, a phenotype which was reverted by both inhibitors. This study provides a preliminary basis for inhibiting endothelial inflammation in vascular inflammatory disorders.

## Highlights

• Use of a high-throughput proteomics inhibitor screen to assess endothelial inflammation
• NFKB or JAK1 inhibition blocked majority of synergetic (TNFα + IFNγ) induced inflammation
• No single inhibitor blocked complete TNFα + IFNγ inflammatory response
• Inflammation-induced VWF release was blocked by NFKB, but not JAK1 inhibition

## Graphical abstract

**Figure:**
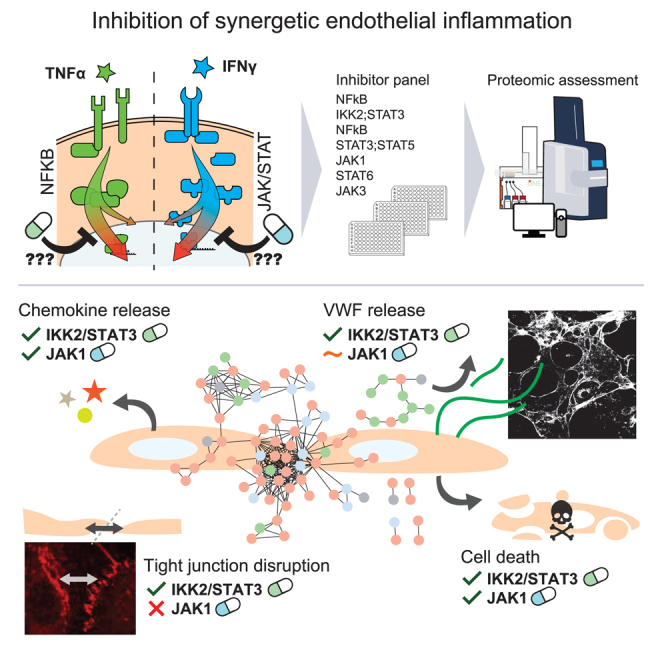

## Introduction

Systemic inflammatory disorders such as Acute Respiratory Distress Syndrome (ARDS), vasculitis, sepsis, and acute cytokine release syndrome (CRS) induced by CAR-T therapies are associated with increased plasma levels of inflammatory stimuli such as cytokines TNFα and IFNγ.^1^^,^^2^^,^^3^^,^^4^ These cytokines promote a pro-thrombotic and pro-inflammatory state of the endothelium, leading to mismanaged platelet interactions, increased leukocyte recruitment and thrombotic events, but no consistently effective treatments are currently available.^5^^,^^6^^,^^7^^,^^8^^,^^9^^,^^10^ Moreover, synergy between TNFα and IFNγ *in vivo* has been linked to inflammatory atherogenesis and the induction of increased cell death in Sars-CoV-2 and Cytokine shock syndromes.^11^^,^^12^ *In vitro* experiments in epithelial and endothelial cells (ECs), suggest this synergy to be driven by the hyperactivation of the JAK-STAT1 axis.^12^^,^^13^^,^^14^ Using *in vitro* models, we have recently also shown that combined exposure of TNFα and IFNγ results in a synergetic hyperinflammatory state in ECs, driven by interplay between JAK/STAT and NFkB pathways.^15^ Therefore, targeting this synergy could effectively inhibit endothelial inflammation in vascular inflammatory disorders. However, how the inhibition of these activation axis attenuates synergetic endothelial inflammation is unclear.

Inhibition of separate TNFα- or IFNγ-induced inflammation has previously been investigated in ECs.^16^^,^^17^^,^^18^^,^^19^ Blocking TNFα-induced kinases AXL and FYN affected the abundance of endothelial inflammation markers, including VCAM1, ICAM1, and IL6, but had a limited effect on leukocyte-endothelial interactions.^16^^,^^17^ In addition, JAK inhibition was able to inhibit IFNγ-induced responses and attenuated TNFα-mediated inflammation through blocking STAT3 activation.^18^^,^^19^^,^^20^ Although providing valuable insights, these studies focus on selected outcomes such as adhesion proteins (e.g., VCAM1, ICAM1) or thrombotic mediators (e.g., F3, PLAU), but do not capture system-wide effects.

Recent advances in high-throughput mass spectrometry proteomic workflows have allowed inhibitor screening using proteomics.^21^^,^^22^^,^^23^ Here, we utilize this approach to assess system-wide, *in vitro* effects of inhibiting the TNFα- and IFNγ-induced synergetic inflammation response in ECs, using a panel of NFKB and JAK/STAT pathway inhibitors. We show that IKK2/STAT3 inhibitor TPCA1 affects both TNFα- and IFNγ-responses, while JAK1 inhibitor itacitinib blocked the IFNγ response. The majority of proteins induced by synergetic inflammation were blocked by both inhibitors, including pyroptosis mediators and chemokines. Moreover, we observed that synergetic inflammation resulted in an extracellular network of VWF, which could be reversed by either inhibitor. Taken together, this study provides molecular insights into treatment avenues targeting endothelial inflammation in vascular inflammatory disorders.

## Results

### System-wide inhibition of inflammation states and steady state effects

**Figure 1:**
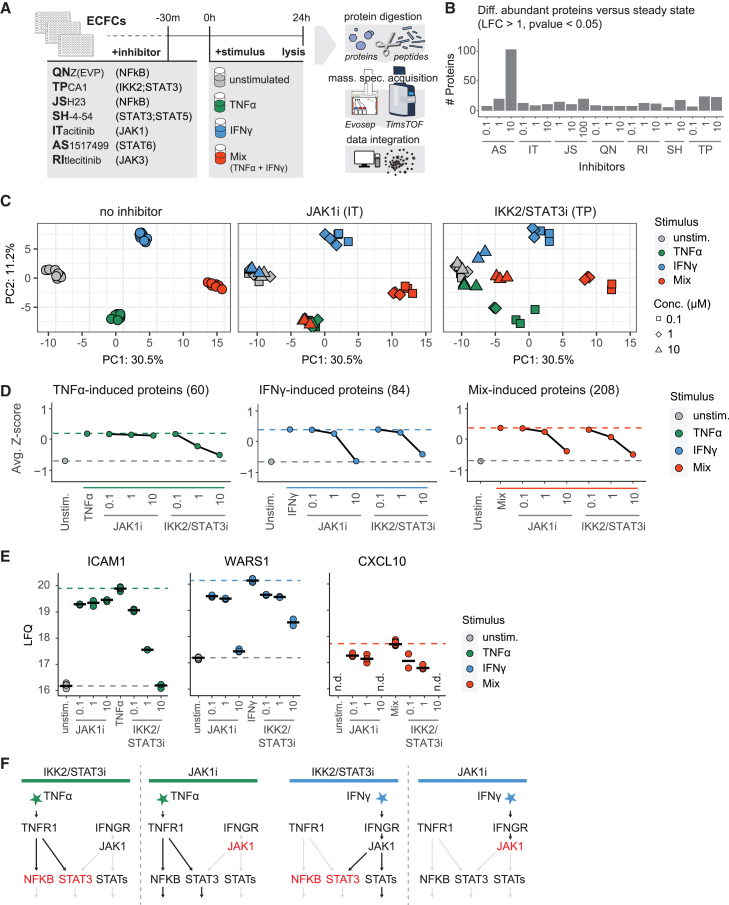
IKK2/STAT3 and JAK1 inhibition affects endothelial inflammation states (A) Overview of inhibitor panel with their targets and proteomics workflow. (B) Differentially abundant proteins of inhibitors versus steady state proteome (LFC >1, BH adjusted *p*-value <0.05, moderated t-test). Mean of *N* = 3 replicates, concentrations are shown in μM. (C) Facetted principal component analysis (PCA) of samples combined with IKK2/STAT3i (TP), JAK1i (IT) or without inhibitor per stimulus as indicated: steady state (gray), TNFα (green), IFNγ (blue), TNFα+IFNγ (red). Shapes indicate concentrations: 0 μM (circle), 0.1 μM (square), 1 μM (diamond), 10 μM (triangle). (D) Mean z-scores of regulated proteins per stimulus and inhibitors as indicated. TNFα-induced (green, *N* = 60 proteins), IFNγ-induced (blue, *N* = 84), Mix-induced (red, *N* = 208). Horizontal lines indicate average *Z* score of conditions without inhibitor. (E) LFQ levels of inflammation proteins across inhibitors and concentrations per stimulus. *n.d.* indicates protein was not detected. Crossbar indicates mean (*N* = 9 biological replicates for stimuli without inhibitors, and *N* = 3 for stimuli with inhibitors). Dotted line shows the mean steady sate or stimulated levels without inhibitors. (F) Schematic overview of pathway inhibition for separate stimuli, showing how JAK1i has no effect on TNFα induced response.

To explore the effect of the inhibition of JAK1/STAT and NFKB pathways on synergetic endothelial inflammation, we selected a panel of 7 inhibitors, consisting of NFKB inhibitors QNZ(EVP4593) and JSH23, IKK2/STAT3 inhibitor TPCA, JAK1 inhibitor Itacitinib, JAK3 inhibitor Ritlecitinib, STAT3/STAT5 inhibitor SH-4-54, and STAT6 inhibitor AS1517499 (Table S1). First, we assessed inhibitor effects on cell viability and EC phenotype. Relative cell viability was reduced 3-fold by incubation with STAT3/STAT5i (100 μM), and this condition was removed from analysis. (Figure S1). Incubation with STAT6i (10 μM) resulted in a disruption of the endothelial monolayer, but cell viability was not decreased (Figure S1). Next, we investigated inhibitor effects on the proteome through a high-throughput workflow, employing label-free-quantification (LFQ), resulting in an average of 5,821 quantified proteins across samples (Figure 1A). Before investigating the inhibition of inflammation, we assessed the effects of the inhibitors on the steady state proteome. STAT6i (10 μM) induced 103 differentially abundant proteins versus the steady state (LFC >1 and *p* < 0.05) (Figure 1B). The highest biological process enrichment of these proteins was “tube morphogenesis,” which included the downregulation of known angiogenic proteins such as KDR,^24^ ROBO4^25^ and ESM1^26^ (Figure S2). Protein changes induced by other inhibitors were limited, ranging from 6 to 24 differentially abundant proteins. As STAT6i (10 μM) had a large effect on the steady state proteome and the EC monolayer, we did not include it in further analysis. Next, we investigated the effect of the inhibitors on synergetic inflammation states. Principal component analysis (PCA) of the TNFα, IFNγ, and TNFα+IFNγ (Mix) inflammation states indicated JAK1i (IT) affected the IFNy response, while IKK2/STAT3i (TP) affected both IFNγ- and TNFα-induced protein regulation (Figure 1C). The other inhibitors of NFKB, STAT3; STAT5i, STAT6i, and JAK3i did not affect inflammation states and were not analyzed further (Figure S3). Averaging protein levels affected per inflammation state demonstrated that TNFα-induced proteins were dose-dependently inhibited by IKK2/STAT3i, while JAK1i had no effect on these proteins (Figure 1D). The IFNγ-induced protein increases were reverted to steady state by JAK1i and reduced to a large extent by IKK2/STAT3i. Interestingly, these effects were only observed at the highest concentration (10 μM) used for both inhibitors. Finally, the proteins induced by combined stimulation were drastically reduced by either inhibitor at the highest concentrations. These signatures were illustrated by hallmark inflammation proteins ICAM1 (TNFα-response), WARS1 (IFNγ-response), and synergistically induced CXCL10 (Mix-response) (Figure 1E). These findings indicate that neither inhibitor blocks both axes completely. JAK1i had no effect on TNFα-induced signaling, which is in agreement with the concept that TNFα does not activate NFKB and STAT3 through JAK1^27^ (Figure 1F). On the other hand, IKK2/STAT3i affected the IFNy response only partially. Although STAT3 is downstream of IFNy-induced JAK1 signaling, multiple other STATs are activated by JAK1 signaling,^1^ which potentially drive the remaining IFNy response.

### IKK2/STAT3 and JAK1 inhibition attenuates synergetic inflammation

**Figure 2:**
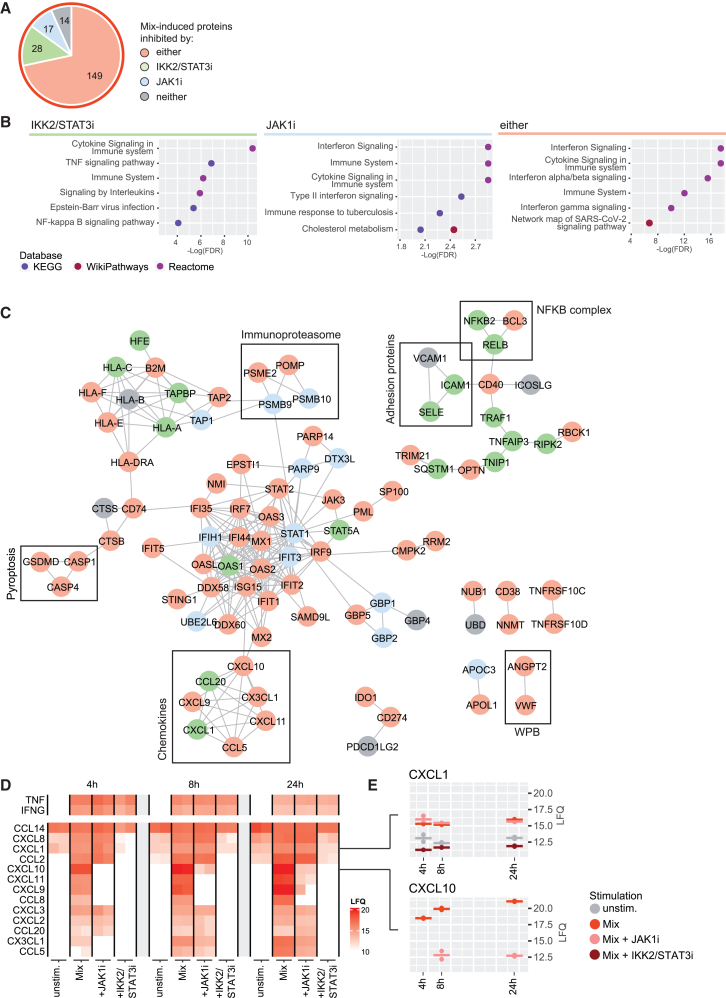
Inhibition of synergetic EC inflammation by IKK2/STAT3i and JAK1i (A) Pie chart of Mix-induced proteins (LFC >1, BH adjusted *p*-value <0.01, moderated t-test) categorized by inhibition: IKK2/STAT3i (TP) (light green), JAK1i (IT) (light blue), either (both TP or IT, light red), or none (gray). (B) Top 6 enriched terms of proteins inhibited by IKK2/STAT3i (green), JAKi (blue) or either inhibitor (red) in KEGG (purple), WikiPathways (dark red), and Reactome (dark purple) databases. (C) Protein interaction network based on the highest confidence (>0.9) StringDB interactions. Processes of interest are indicated in squares. (D) Heatmap of chemokine LFQ levels in the secretome at 4h, 8h, and 24h in conditions as indicated. TNFα and IFNγ are added experimentally. Color indicates conditions, unstimulated (gray), Mix (red), Mix + JAKi (pink), Mix + IKK2/STAT3i (dark red). (E) LFQ levels of chemokines CXCL1 and CXCL10 in conditions as indicated. Crossbar indicates mean (*N* = 2 biological replicates).

Next, we further investigated the inhibition of the combined inflammation state. The majority of the 208 Mix-induced proteins were inhibited by both inhibitors (149 proteins), reiterating the synergetic dependency on both JAK/STAT and NFKB activation for a major part of this endothelium inflammation state (Figure 2A). 28 proteins were inhibited by IKK2/STATi only, 17 by JAK1i only, and 14 proteins that remained significantly regulated regardless of inhibition. We analyzed the associated pathways by performing pathway-enrichment analysis of KEGG, Reactome and WikiPathways databases. As expected, this indicated that proteins inhibited by IKK2/STAT3i were enriched for TNF-signaling and NFKB-signaling, while proteins inhibited by JAKi were enriched for (Type II) interferon signaling (Figure 2B). Interestingly, proteins inhibited by both inhibitors, enriched for interferon signaling as well, specifically for interferon alpha/beta and for SARS-CoV-2 signaling network. A StringDB interaction network (high confidence interactions) revealed more specific processes affected by each inhibitor (Figure 2C). The upregulation of the NFKB complex and adhesion proteins were only inhibited by IKK2/STAT3i, while members of the immunoproteasome were inhibited by JAK1i. Protein expression blocked by both inhibitors included pyroptosis mediators (GSDMD, CASP1, and CASP4), chemokines (CXCL9/10/11 and CCL5), and Weibel Palade body (WPB) content (VWF and ANGPT2), indicating the synergetic induction of these proteins. Finally, proteins that were incompletely inhibited by either inhibitor included membrane proteins VCAM1, PDCD1LG2 (CD273), and ICOSLG (Figure S4). Prompted by the upregulation of pyroptosis proteins, we analyzed changes in the number of cell deaths by combined stimulation and inhibition. Combined stimulation resulted in a significant (*p* < 0.05), but modest increase in cell death (from 4% to 12% on average), which was reverted by the inhibition of both JAK1i and IKK2/STAT3i (Figure S5). To assess secreted chemokines and time-dependent changes in protein abundance profiles, we analyzed secretomes and lysates 4h, 8h, and 24h after stimulation with TNFα and IFNγ in combination with IKK2/STAT3 or JAK1 inhibition. Both lysates and secretomes showed time-dependent inflammation signatures with the largest differences after 24h, although secretome clustering was less distinct (Figure S6A). Interestingly, IKK2/STAT3i completely blocked the overall Mix-response at 4h and 8h (0% and 2% of Mix-induced proteins remained significant), while JAKi blocked an equal part of protein induction at all timepoints (39%, 41% and 39% remained significant at 4, 8 and 24h respectively) (Figure S6B). Investigating the released chemokines specifically showed that the majority of Mix-induced chemokines were downregulated by both JAK1i and IKK2/STAT3 (e.g., CXCL10), while some were only inhibited by IKK2/STAT3i (e.g., CXCL1), in line with observations in the lysates (Figures 2D and 2E). These findings indicate that the inhibition of the NFKB or JAK/STAT axis blocks the majority of proteins induced by combined stimulation, but that neither inhibitor blocks TNFα- or IFNγ-induced signaling completely.

### Inhibition of synergetic Von Willebrand Factor and tight-junction regulation

**Figure 3:**
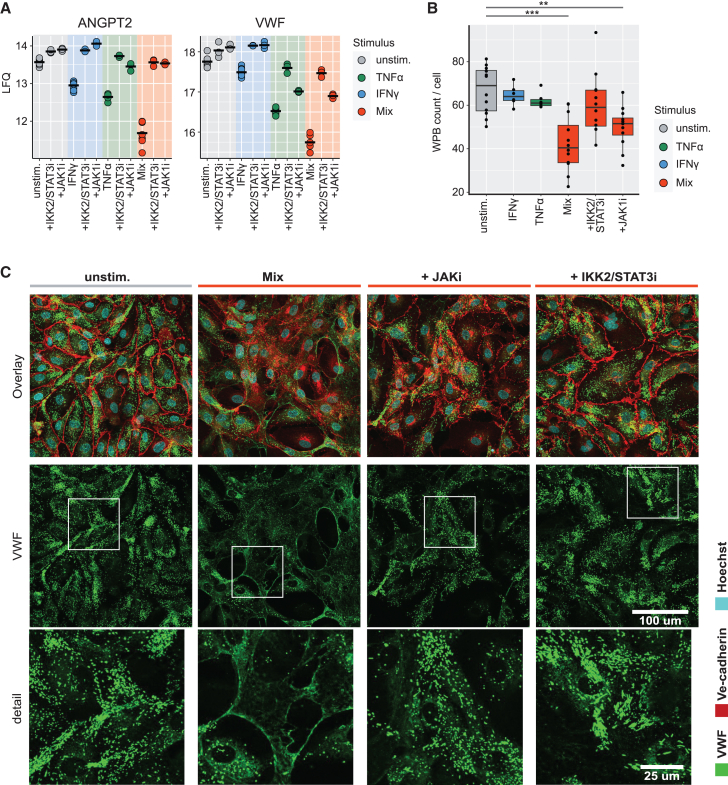
Inhibition of inflammation-induced VWF release (A) LFQ levels of WPB proteins per inhibitor (10 μM) and stimulus as indicated: steady state (gray), TNFα (green), IFNγ (blue), TNFα+IFNγ (red). Crossbar indicates mean (*N* = 9 biological replicates for stimuli without inhibitors, and *N* = 3 for stimuli with inhibitors). (B) Quantified WPB count per cell from confocal imaging in conditions as indicated. Boxplot middle line indicates median, upper and lower parts of boxplots indicate the 25th and 75th percentiles (*N* = 4 independent experiments for unstimulated and mix conditions, *N* = 2 independent experiments for TNFα and IFNγ stimulated conditions, 3 images were analyzed per experiment). Significant differences are indicated: ∗∗ = adj. *p*-value <0.01, ∗∗∗ = adj. *p*-value <0.0001. (one-way-Anova and Tukey post-hoc test). (C) Confocal images of ECs stained with anti-VWF (green) and anti-Ve-cadherin (red) antibodies in conditions as indicated. Scale bar (white) indicates 100 μm for overall image and 25 μm for the detail images. Brightness and contrast were adjusted equally across images, representative image shown (*N* = 4 independent experiments, 3 images were analyzed per experiment).

We further investigated ANGPT2 and VWF, two important endothelial dysfunction markers in multiple inflammatory disorders.^28^^,^^29^ Protein levels were reduced upon inflammation, which was reverted to steady state levels with IKK2/STAT3i or JAK1i for ANPGT2, and partly reverted for VWF (Figure 3A). Next, we imaged VWF in combination with EC tight-junction protein VE-cadherin (CDH5) in inflamed endothelial monolayers. Strikingly, after combined stimulation, ECs had reduced amounts of WBPs (average of 40 WPBs per cell versus 67 in the unstimulated controls, *p* < 0.001) (Figure 3B). This decrease was rescued partly in combination with JAK1i (50 WPBs/cell, *p* < 0.01) and returned to baseline levels with IKK2/STAT3i (60 WPBs/cell, not significant). Moreover, after combined stimulation, VWF was visible as an extracellular network, which was not visible in combination with either inhibitor (Figures 3C and S7). Interestingly, VWF levels in the secretomes were modestly increased by combined stimulation (log fold change 0.6–0.7 versus unstimulated controls at all timepoints) (Figure S8A). Validation by ELISA also showed a non-significant increase in Mix-stimulated cells compared to unstimulated controls (0.04–0.05 UI/ml respectively), which returned to baseline with IKK2/STAT3i, but not JAKi (Figure S8B). Next to affecting VWF, inflammation induced the disruption of tight-junctions, which was mostly blocked with IKK2/STAT3i, but to a lesser extent with JAK1i, based on confocal images and changes in average junction width and length (Figures S9A and S9B). Interestingly, TNFα induced a similar reduction in width and length as combined stimulation, while IFNγ caused a slight increase in junction width (Figure S9B). To gain an indication of how the EC barrier function was affected, we performed an Electric cell-substrate impedance sensing (ECIS) assay. In line with observations on VE-Cadherin disruption, combined stimulation induced a decrease (50%) in resistance regardless of combination with JAKi, which did not recover over 6 h (Figures S9C and S9D). On the other hand, IKK2/STAT3i resistance levels were similar to unstimulated control. As such, we show the effect of synergetic inflammation on VWF and tight-junctions in ECs, which could be attenuated by the inhibition of both IKK2/STAT3i and, to a lesser extent, with JAK1i. Taken together, these findings demonstrate the potential of inhibiting synergetic EC inflammation by targeting the NKFB and JAK/STAT activation axis.

## Discussion

In this study, we dissect the inhibition of synergetic endothelial inflammation by pharmacological intervention in the JAK/STAT and NFKB pathways. Employing proteomics, we demonstrate the system-wide inhibition of inflammation-induced proteins by JAK1 inhibitor itacitinib and IKK2/STAT3 inhibitor TPCA1, in proteins associated with VWF release, pyroptosis, and chemokines.

Synergism between TNFα and IFNγ has been shown to contribute to excessive inflammation and organ damage in SARS-COV-2 and Cytokine Shock Syndromes driven by the hyperactivation of the JAK/STAT1/IRF1 pathway.^12^^,^^13^^,^^14^ Here, we show synergistically regulated proteins that are blocked by inhibiting either the NFKB or JAK/STAT activation axis. Among these were inflammasome proteins CASP1, CASP4 and GSDMD, which mediate pyroptosis.^30^^,^^31^ This process has been indicated to drive synergistically induced inflammatory cell death in mice,^12^ intestinal epithelial cells^14^ and corneal cells.^32^ We also observe the synergetic upregulation of these proteins and demonstrate that both JAK1 and IKK2/STAT3 inhibition blocks their expression and increased cell death. Another group of important synergistically induced proteins was chemokines, among which CCL5 and CXCL10.^15^^,^^33^^,^^34^ CCL5 has been shown to facilitate (CCR5-mediated) interactions in multiple inflammatory disorders, and both chemokines are suggested as targets for treating CRS, graft-versus-host disease, and rheumatoid arthritis among others.^35^^,^^36^^,^^37^ We show that the endothelial expression of both CCL5 and CXCL10 can be blocked by inhibiting either NFKB or JAK activation. Finally, VWF and ANGPT2 plasma levels are used as markers for endothelial dysfunction in inflammatory disorders.^28^^,^^29^ It is known that inflammatory triggers such as TNFα induce VWF release,^38^ however, as we show here, it is amplified in combination with IFNγ. TNFα + IFNγ synergy resulted in a drastic reduction in WPBs, which was reversed by blocking IKK2/STAT3 and, to a lesser extent, JAK1. Inflammation-induced disruption of tight junctions was also blocked in combination with IKK2/STAT3i, but not JAK1i. Moreover, the inflammatory response 4 and 8 h after TNFα+IFNγ stimulation was completely blocked by IKK2/STAT3i, but not by JAK1i. These findings suggest TNFα signaling through NFKB induces a more immediate response, both in the disruption of tight junctions and the early transcription of new proteins. On the other hand, the IFNγ-response increases steadily over 24 h, suggesting the dynamics of transcription factor activation and prolongation are different between both cytokines in line with previous findings.^15^^,^^18^^,^^39^

In contrast, extracellular VWF strings induced by combined stimulation were not visible in combination with both IKK2/STAT3i and JAK1i. Whether this is an artifact of *in vitro* culturing or has dedicated biological functions, similar to the formation of neutrophil extracellular traps (NETs), for example, remains to be investigated. However, these observations provide a mechanistic basis for increased levels of VWF by synergetic TNFα and IFNγ endothelial inflammation.

Combining these findings suggests that targeting the NFKB and JAK/STAT activation axis can block important processes in synergistically inflamed ECs and could provide merit in vascular inflammatory disorders. Itacitinib has indeed been assessed in phase I and II studies on reducing CAR-T induced cytokine shock syndrome CRS,^40^^,^^41^ which is associated with elevated levels of TNFα and IFNγ.^2^ However, in our study, JAK1 inhibition did not affect proteins that are uniquely expressed by TNFα exposure, among which important leukocyte interactors such as adhesion protein VCAM1 and T cell receptor ICOSLG. Moreover, tight junctions remained disrupted in combination with itacitinib. On the other hand, TPCA1, which inhibits both NFKB activation and STAT3,^42^ blocked the TNFα response and the majority of the IFNγ response. Our results therefore demonstrate that targeting a single axis inhibits a large part, but does not completely block endothelial inflammation induced by combined exposure *in vitro*.

Whether targeting a single axis is sufficient to block synergetic vascular inflammation *in vivo* remains to be investigated. However, our findings emphasize the importance of studying cytokine combinations when investigating treatment options in disorders with elevated levels of multiple inflammatory stimuli. In conclusion, this study highlights the use of a proteomic inhibitor screen to reveal the inhibition of TNFα- and IFNγ-induced synergy in ECs by targeting the NFKB and JAK/STAT activation pathway. It highlights the dependency on both axes to induce synergetic inflammation and highlights the link between inflammation and VWF release. As such, this study provides an *in vitro* based assessment of targeting synergetic endothelial inflammation by inhibiting NFKB and JAK/STAT activation.

### Limitations of the study

These findings are based on an *in vitro* model, and we should consider that this setting lacks dynamic factors such as flow, vessel size and organ heterogeneity that might affect inflammatory responses.^43^^,^^44^^,^^45^^,^^46^^,^^47^^,^^48^ Moreover, although we have previously observed highly conserved inflammatory responses in different donors *in vitro*,^15^^,^^44^ inflammation responses and inhibition dynamics vary from person-to-person *in vivo*. Potential donor-, sex- and/or gender-specific differences can therefore not be assessed in this *in vitro* setting. In this study, we focused on the inhibition of the combined TNFα and IFNγ response. The next step in dissecting the underlying drivers of cytokine synergy will require a combination of (short)-term phospho-signaling experiments, dedicated gene editing of proteins of interest, or a more extensive panel of inhibitors that targets all kinases and transcription factors of both pathways. Finally, this study focuses on the endothelial response, while inflammatory stimuli in systemic disorders will affect multiple cell types, such as platelets and leukocytes, that we do not assess here.

## Resource availability

### Lead contact

Further information and requests for resources and reagents should be directed to and will be fulfilled by the Maartje van den Biggelaar (m.vandenbiggelaar@sanquin.nl).

### Materials availability

This study did not generate new unique reagents.

### Data and code availability

• The raw mass spectrometry acquisition files and DIA-NN search files have been deposited to the ProteomeXchange Consortium via the PRIDE^49^ partner repository with dataset identifier PXD058294 (inhibitor panel screen) and PXD064921 (Early timepoint lysates and secretomes). Analyzed data used for figures in this study can be found in Data S1.
• No original code was generated in this study.
• Any additional information required to reanalyze the data reported in this article is available from the lead contact upon reasonable request.

## Acknowledgments

This work was supported by the 10.13039/100009425Landsteiner Foundation for Blood Transfusion Research grants LSBR-1517 awarded to M.v.d.B. and LSBR-1923 awarded to A.J.H. and M.v.d.B.

## Author contributions

S.A.G. designed the study, performed experiments, analyzed data, and drafted the article. P.L. developed the mass spectrometry workflow and contributed to data acquisition. G.M. and A.B. performed experiments and analyzed data. M.v.d.B. and A.J.H. designed the study, supervised research, and drafted the article. All authors read and approved the final version of the article.

## Declaration of interests

The authors declare no competing interests.

## STAR★Methods

### Key resources table

**Table:** 

REAGENT or RESOURCE	SOURCE	IDENTIFIER
**Antibodies**		

mouse-monoclonal anti-VWF (RAG20)	Sanquin^50^	N/A
goat polyclonal anti–VE-cadherin	Santa-Cruz	CAT# sc-6458; Lot# H1814; RRID: AB_2077955
mouse-monoclonal anti-VWF IgG mix for ELISA	Sanquin^50^	N/A

**Chemicals, peptides, and recombinant proteins**		

Endothelial Growth Medium, Ready-to-use	PromoCell	Cat# C-22011
Inhibitors	Selleck Chemicals	See Table S1
IFNγ	Peprotech	Cat# 300-02-20UG; Lot# 102227 K0822
TNFα	Peprotech	Cat# 300-01A-10UG; Lot# 031825 H2322

**Critical commercial assays**		

CellTiter-Blue Viability Assay	Promega	G8080
LIVE/DEAD™ Fixable Near-IR Dead Cell Stain Kit, for 633 or 635 nm excitation	Thermo Fisher	L34975

**Deposited data**		

Mass spectrometry raw files	This paper	PXD: PXD058294; PXD: PXD064921
DIA-NN analysis files	This paper	PXD: PXD058294; PXD: PXD064921

**Experimental models: Cell lines**		

Endothelial colony forming cells	Isolated in house^51^	N/A

**Software and algorithms**		

DIA-NN v1.8.1	Demichev et al.^52^	https://github.com/vdemichev/DiaNN
R v4.2.3	R Core Team (2023)	https://r-project.org/
R/Studio release 2022.07.02	Posit team (2025)	https://posit.co/
Cytoscape v3.10.0	Shannon et al.^53^	https://cytoscape.org/
Fiji v2.9.0	Schindelin et al.^54^	https://imagej.net/software/fiji/downloads
CellProfiler v4.2.8	Stirling et al.^55^	https://cellprofiler.org/
CellProfiler OrganelleProfiler pipeline	Laan et al.^56^	https://doi.org/10.1371/journal.pone.0278009.s005

### Experimental model and study participant details

Endothelial colony forming cells (ECFCs), were isolated from healthy donors as described by Ramirez et al.,^51^ in accordance with Dutch regulations and the Declaration of Helsinki after approval from the Sanquin Ethical Advisory Board. Written informed consent was given by all participants. Cells tested negative for mycoplasma contamination and expressed endothelial markers VWF and VE-Cadherin. ECFCs from three different donors (2x F, 1x M) were combined for assays. Culture flasks were coated with collagen type I (50 μg/ml, BD biosciences) prior to use. Cells were cultured in endothelial cell basal medium (Lonza) supplemented with 18% FCS (Bodinco) and EGM bulletkit (Lonza) at 37^o^C and 5% CO_2_.

#### Stimulation

Inhibitors were obtained from SelleckChem, see Table S1. Cell viability in combination with inhibitors was assessed for 24h using the CellTiter-Blue cell viability assay (Promega), according to manufacturer’s instructions. Cytokines were obtained from Peprotech. For proteome analysis, cells were seeded in three 96-well plates. Inhibitors or DMSO controls were added at indicated concentrations (0.1-1-10 μM, or 1-10-100 μM), 30 minutes prior to cytokine stimulation. Subsequently, either 10 ng/ml TNFα, IFNγ, TNFα+IFNγ was added and cells were harvested after 24h. All stimulation with inhibitors were performed in triplicate, and conditions without inhibitor, in 9 replicates (3 per plate).

#### Flow cytometry cell death assay

For the cell death assay ECs were stimulated in triplicate with inhibitors and cytokines as indicated for 24h. Supernatant and ECs detached with trypsin were collected and pooled for each condition. Cells were washed three times with PBS and then stained at 4°C for 30 minutes with 1:1000 LIVE/DEAD Fixable Near-IR Dead Cell Stain (Thermo Fisher Scientific). Subsequently, samples were washed three times with PBS and loaded in a BD FACSCanto II Flow Cytometer (BD biosciences) for analysis. Flow cytometry data was analyzed utilizing the FlowJo v10.10.0 software. Statistical comparisons of stimulated conditions versus control were conducted using Kruskal Wallis and post-hoc Dunn’s test. P-value < 0.01 was considered significant.

#### Immunofluorescence

ECFCs were grown to confluence on collagen-coated glass coverslips. After four days, cells were stimulated with 10 ng/ml TNFα+IFNγ in combination with 10 uM Itacitinib or 10 uM TPCA-1, or were not stimulated. Slides were prepared and staining was performed as described previously,^57^ using mouse-monoclonal anti-VWF (RAG20) (Sanquin, 10 μg/mL)^50^ and goat polyclonal anti–VE-cadherin antibodies (Santa-Cruz, sc-6458, #H1814, 1 μg/mL). An SP8 confocal laser scanning microscope (Leica) with a 63x/1.30 oil objective was used to acquire images. Images were processed using Fiji (2.9.0),^54^ brightness and contrast were adjusted for visualization purposes. For threshold images, thresholds were set equally across images per experiment using the Huang method.^58^ Quantification of WPB counts was performed using Cell Profiler (4.2.8),^55^ employing the pipeline to quantify WBPs developed by Laan et al.^56^ WPB object area was filtered on size (Area Shape < 50) to limit quantification of strings as WPBs. From this pipeline we generated a new workflow to quantify membrane objects (Data S1). Statistical comparisons between stimulated conditions and unstimulated cells were done using a one-way-Anova and a Tukey post-hoc test, adjusted p-values < 0.05 were considered significant. N = 4 independent experiments were performed for unstimulated and mix conditions and N = 2 experiments for TNFα and IFNγ stimulated conditions. 3 images were analyzed per experiment.

#### VWF ELISA

ECFCs were stimulated with 10 ng/ml TNFα+IFNγ in combination with 10 uM Itacitinib or 10 uM TPCA-1, or were not stimulated. After 24h supernatant was collected. For the ELISA, microtiter plates were coated with 5 ug/ml anti-VWF IgG mix (Sanquin^50^) in 50mM NaHCO3 pH 9 (Sigma Aldrich). Plates were incubated overnight at 4^o^C and washed 5x in PBS (Fresenius Kabi) – 0.1% Tween20 (Sigma Aldrich) using the Skan Washer 400 (Molecular Devices). All subsequent wash steps were performed as outlined here. Wells were then blocked with 1% BSA (Serva) in PBS-0.1% Tween20 for 1 h at 37°C. After washing, samples and human plasma standard (1.21IU/mL, Sanquin) were diluted in 1% BSA PBS-0.1% Tween20 and loaded. Plates were incubated at 37^o^C for 1h. Plates were then washed and incubated with VWF-HRP (1.3g/L, Agilent/Dako, P022602, lot# 00095439) diluted 2000x in 1% BSA PBS-0.1% Tween20. After 1h at 37^o^C, plates were washed and developed with 1.1M NaOAc (Sigma Aldrich), 2mg/mL TMB (Sigma Aldrich) in DMSO (Sigma, Aldrich), and 1.5% H2O2 (Sigma Aldrich). Development was halted with 1M H2SO4 (VWR Chemicals). Plates were measured with the Spectra Max Plus 384 (Molecular Devices) at 450nm with 620nm as a reference wavelength. Differences between stimulated and unstimulated cells (N = 3 biological replicates) were analyzed using a Mann-Whitney statistical test in which an adjusted p-value < 0.05 was considered significant.

#### Electric cell-substrate impedance sensing

Seeding the Electric Cell-substrate Impedance Sensing (ECIS) arrays and monitoring of the Trans-endothelial electrical resistance (TER) was performed as described previously.^59^ After forming a stable monolayer, cells were stimulated with 10 ng/ml TNFα+IFNγ in combination with 10 μM itacitinib (JAK1i), 10 μM TPCA-1 (IKK2/STAT3i) or without inhibitor. Unstimulated cells were mock stimulated with DMSO. All conditions were performed in N = 2 replicates. TER was monitored was monitored for 6 h at a frequency of 3,000 Hz and a measurement interval of 60 s. Resistance values were normalized to the average resistance 1.5 h before addition of stimuli per condition.

#### Mass spectrometry analysis

For mass spectrometry analysis of EC proteomes, cells were lysed and proteins were digested with trypsin as described previously.^44^ Secretomes were collected prior to cell lysis and spun down at 5,000 g to remove cell debris. Continued processing was the same as for lysates, but SDC was omitted from the lysis buffer. Tryptic digests were transferred to an Evotip Pure (Evosep) according to manufactures guidelines and separated on a 8 cm × 150 μm, 1.5 μm Performance Column (EV1137 from EvoSep) with an Evosep One liquid chromatography (LC) system (Evosep) using the 60 samples per day gradient. The lysates and secretomes for 4,8, 24h time point experiment was analyzed using the 30 samples per day gradient. Buffer A was composed of 0.1 % formic acid, buffer B of 0.1 % formic acid in acetonitrile (Biosolve, NLD). Peptides were ionized and electro sprayed into a TimsTOF HT mass spectrometer (Bruker). Data was acquired in diaPASEF mode, using an MS1 scan range of 100-1700 m/z. For MS2 acquisition 32 pyDIAID system-optimized^60^ windows were used, with a cycle time of 1.80 seconds and a mass and ion mobility range from 400.2-1500.8 m/z and 0.70-1.50 1/k0 respectively. Collision energy used was 20.00 eV at 0.6 1/k0 and 59 eV at 1.60 1/k0.

#### Data analysis

Raw mass spectrometry data files were processed using DIA-NN software (version 1.8.1),^52^ proteins and peptides were detected by querying the filtered human Swissprot database (release 2021.22.04). Standard settings were used, using a generated library-based spectra search. Maximum number of variable modifications was set at 2, protein interference as “Protein names (from FASTA)” and quantification strategy as “Robust LC (high accuracy).” DIA-NN protein quantification result files were analysed using R 4.2.3/Rstudio (2022.07.02) as follows. Detected proteins were filtered for proteotypic peptides. Plasma contaminants were removed and proteins were filtered based on detection in at least one (un)stimulated condition without inhibitors (9 replicates). Eight samples with a deviation of two times the standard deviation from the mean number of proteins quantified were omitted from analysis. LFQ values were transformed in log2 scale. Missing values were imputed by normal distribution (width = 0.3, shift = 1.8), assuming these proteins were close to the detection limit. Label free statistical analyses were performed using LIMMA,^61^ employing moderated t-tests to determine differentially abundant proteins. A Benjamin Hochberg-adjusted p value < 0.01 and log2 fold change > 1 was used as significance threshold. Gene ontology term enrichment and pathway analyses were performed using StringDB,^62^ enrichments with a BH-adjusted p-value <0.05 were considered significant. Interaction networks were based on String database interactions and were visualized in Cytoscape 3.10.0, using th e “Edge-weighted Spring Embedded Layout” to visualize the network.

### Method details

#### ECFC isolation

Whole blood was diluted 1:1 in PBS and layered on top of a Ficoll-Paque PLUS (Merck) solution prior to centrifugation for 20 min at 1,000 g. After this, the buffy coat was collected and spun down two times for 7 min at 540 g, once with PBS and once with EC medium (endothelial cell basal medium (Lonza) supplemented with 18% FCS (Bodinco) and EGM bulletkit (Lonza)). Next, the cell pellet was dissolved in EC medium and distributed over a collagen-coated (50 μg/ml, BD biosciences) 48 well plate. The cells were grown at 37°C and 5% CO_2_ until colonies appear (14 – 28 days), refreshing the EC medium three times per week. Once colonies become confluent, cells were expanded into a 24- and 6-well plate, and finally a T75 flask. At this stage cell expression of endothelial markers VWF and VE-cadherin was assessed using immunofluorescence staining. ECFCs are then cryopreserved until further use. For a full step-by-step description of the protocol see Ramirez et al.^51^

#### Immunofluorescence slide preparation and staining

After stimulation, cells were fixed with 4% paraformaldehyde (Thermo Fisher Scientific) and washed 3× with PBS, prior to storage in PBS at 4°C. For staining, coverslips were first quenched with 50 mM ammonium chloride (Sigma Aldrich) and consequently blocked in 1% bovine serum albumin (Serva) and 0.1% saponin (Sigma Aldrich) in PBS. All antibody staining steps were performed in this blocking buffer. Secondary staining for VWF and VE-cadherin were performed with Alexa Fluor 488 chicken-anti-mouse (Invitrogen, #A21200) and Alexa Fluor 568 donkey anti-goat (Invitrogen, #A11057) conjugated secondary antibodies. Slides were fixed in Mowiol 4-88 (Polysciences) and stored overnight at room temperature, prior to confocal microscopy analysis.

#### ECIS array preparation

ECFCs were seeded in 8-well electrode arrays (8W10E, Applied Biophysics) pre-coated with collagen (50 μg/ml, BD biosciences). After seeding, arrays were transferred to a Electric Cell-substrate Impedance Sensing system model 9600 (Applied Biophysics) in an incubator at 37°C and 5% CO_2_. From seeding, trans-endothelial electrical resistance (TER) was monitored every minute at 3,000 Hz. Stabilization of TER indicates the formation of a stable monolayer (+/- 12 h).

#### Mass spectrometry sample preparation and digestion

For preparation of samples for mass spectrometry analysis, cells were lysed lysis buffer containing 1% sodium deoxycholate (Bioworld), 10 mM TCEP (Thermo Scientific), 40 mM chloroacetamide (Sigma-Aldrich) and 100 mM Tris-HCl pH 8.0 (Gibco). Lysates were incubated for 5 min at 95°C and sonicated for 10 min in a sonifier bath (Branson model 2510), after which trypsin gold (Promega) was added in a 1:50 (w/w) protein ratio and digested overnight at room temperature.

### Quantification and statistical analysis

All statistical tests, significance cut-off values, software used and number of experiments and replicates are indicated in the STAR Methods section per experiment and in the corresponding figure legends.

## Supplemental information

Document S1. Figures S1–S9 and Table S1

Data S1. Proteomics and imaging analysis output all figures in this paper are based on
