# Supplementary material for: Targeting synergetic endothelial inflammation by inhibiting NFKB and JAK-STAT pathways
Source: iScience. 2025 Aug 7;28(9):113307. doi: 10.1016/j.isci.2025.113307 (PMC12396245; doi:10.1016/j.isci.2025.113307)
Supplement: Document S1. Figures S1–S9 and Table S1 [file mmc1.pdf]

iScience, Volume *28*

Supplemental information

Targeting synergetic endothelial inflammation by inhibiting NFKB and JAK-STAT pathways

Stijn A. Groten, Pieter Langerhorst, Georgios Malamas, Alastair Barraclough, Arie J. Hoogendijk, and Maartje van den Biggelaar

Supplemental Figures

Supplemental Figure 1

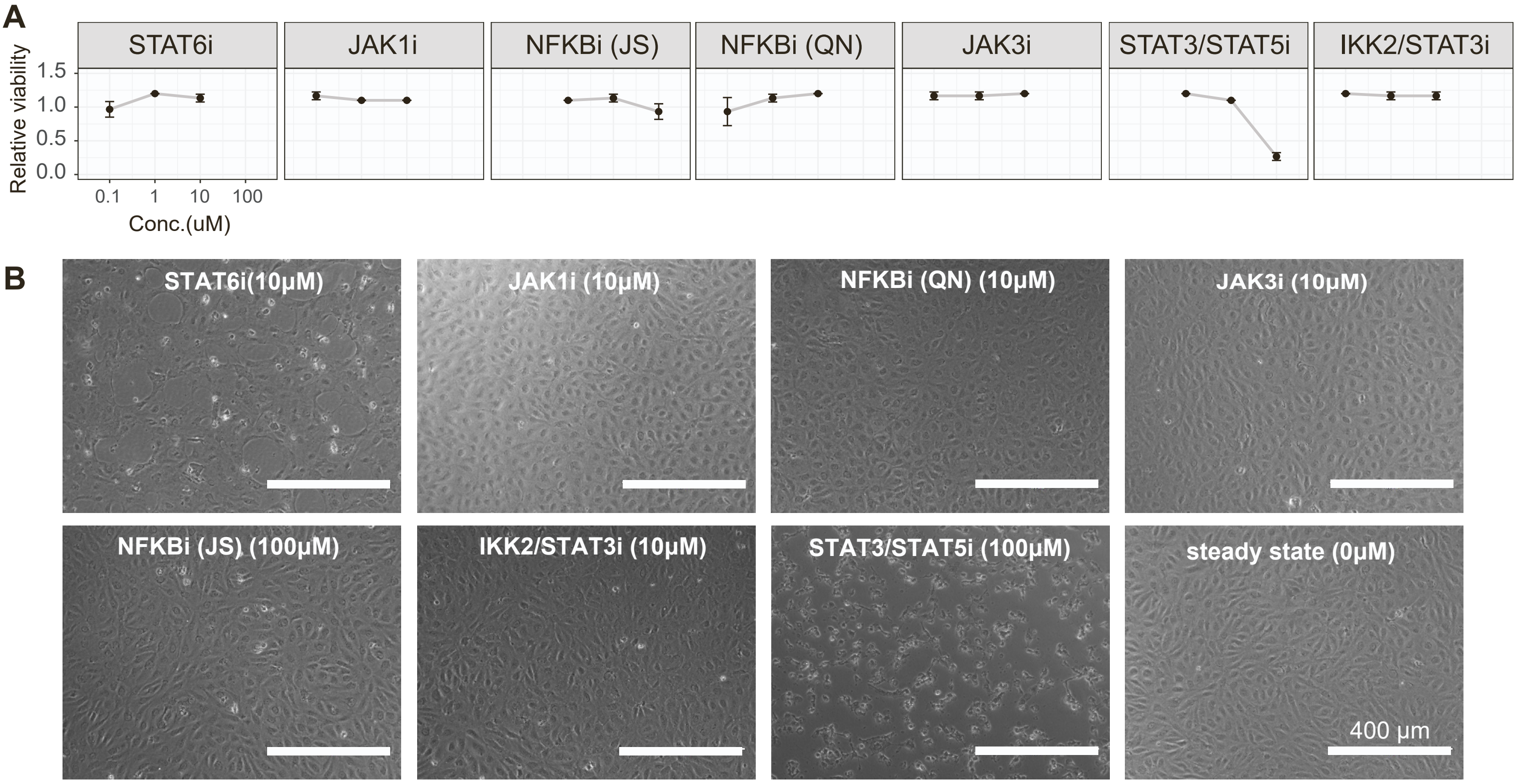

Supplemental Figure 1 Inhibitor effects on endothelial cell viability. A) Relative cell viability of ECs with inhibitor for 24h at concentrations as indicated. Data are represented as mean +/- SD (N = 3 biological replicates per conditions) B) Widefield images of EC monolayers after 24h at highest inhibitor concentration used. Scale bar in white indicates 400 um for all images.

Supplemental Figure 2

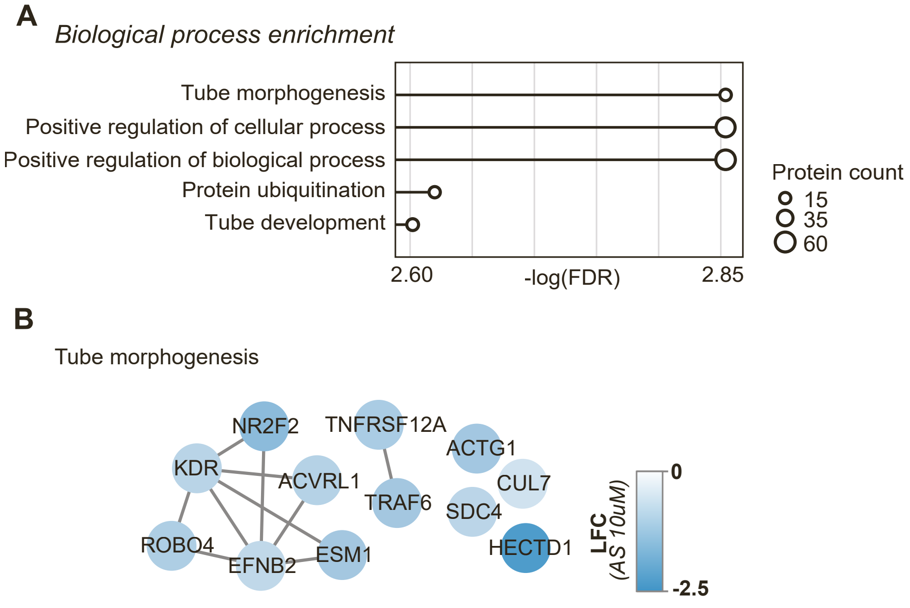

Supplemental Figure 2 Effects of STAT6 inhibition on steady state endothelial cells. A) GO-enrichment of biological process of proteins regualted by AS 10 uM versus steady state. Top five processes shown. B) Protein network of tube morphogenesis term. Color gradient indicates log fold change (LFC) versus unstimulated control. Medium confidence StringDB interactions shown.

Supplemental Figure 3

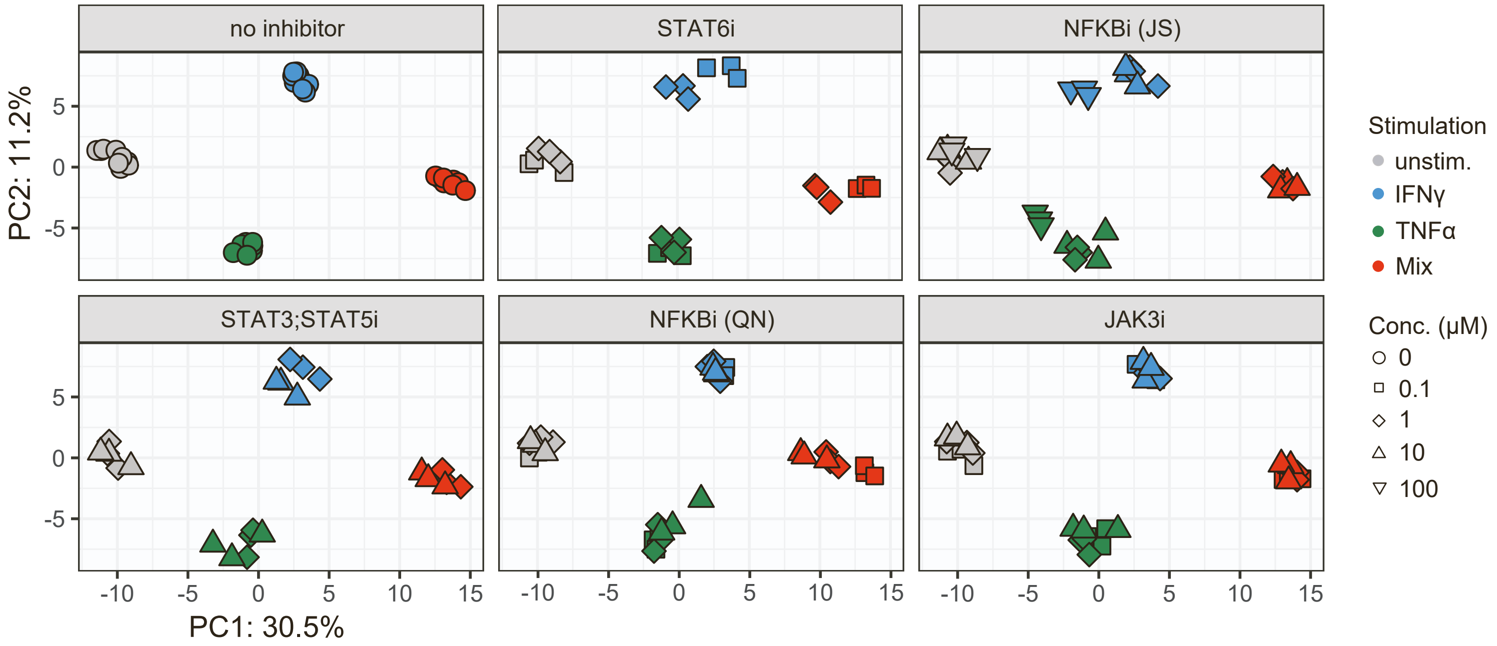

Supplemental Figure 3 Inhibitors with no/limited effect on endothelial inflammation. Facetted PCA of ECs with different stimuli and inhibitor concentrations as indicated: unstimulated (grey), IFNg (blue), TNFa (green), Mix (red). Shapes indicate concentrations: 0 uM (circle), 0.1 uM (square), 1 uM (diamond), 10 uM (triangle), 100 uM (upside down triangle).

Supplemental Figure 4

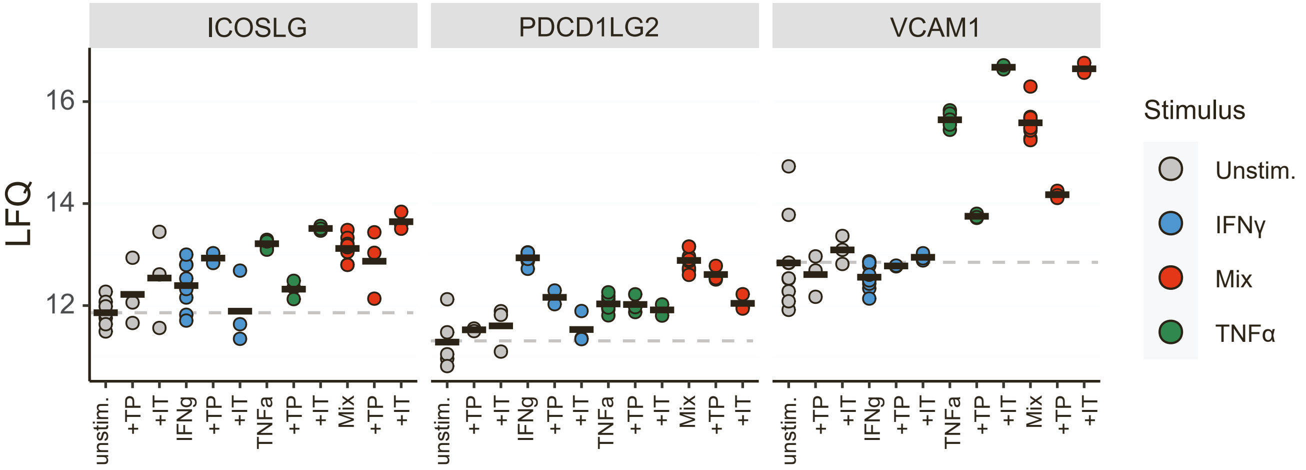

Supplemental Figure 5 Inflammation induced proteins not inhibited by either IKK2/STATi or JAK1i. Dot plot of LFQ levels of proteins. Stimuli are shown as indicated: unstimulated (grey), IFNg (blue), TNFa (green), Mix (red). N = 9 biological replicates for stimuli without inhibitors, and N = 3 for stimuli with inhibitors, crossbar indicates mean. Dashed line indicates mean of unstimulated ECs.

Supplemental Figure 5

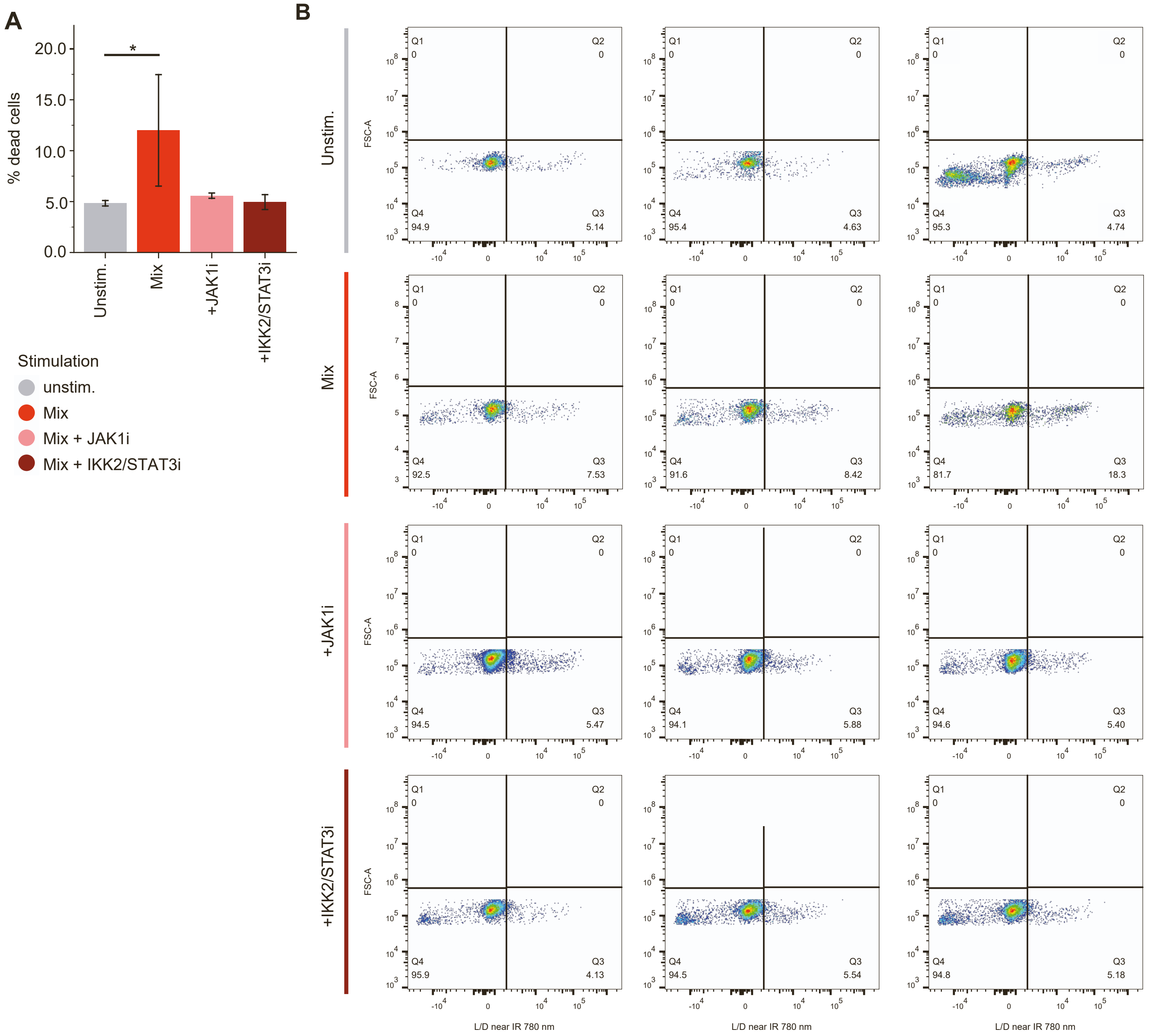

Supplemental Figure 5 JAK1i and IKK2/STAT3i block Mix-induced cell death. A) Bar plots of percentage of death cells per condition as indicated. significant differences indicated: * = adj. p-value < 0.05 (Kruskal Wallis and post-hoc Dunn's test). Color indicates conditions, unstimulated (grey), Mix (red), Mix + JAKi (pink), Mix + IKK2/STAT3i (darkred). Data are represented as mean +/- SD (N = 3 biological replicates) B) Flow cytometry measurements in different conditions with plots of forward scatter (FSC-A) and live/dead stain.

Supplemental Figure 6

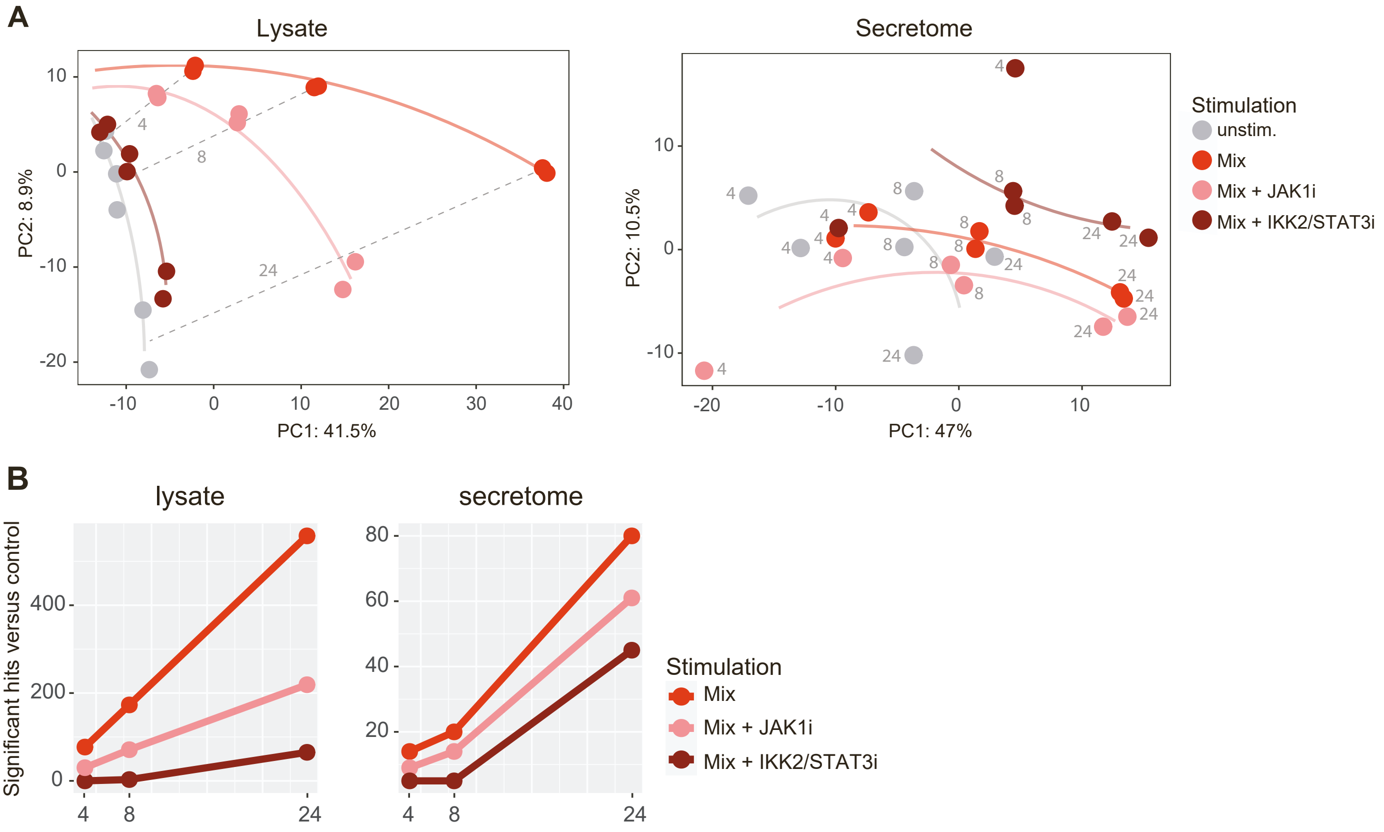

Supplemental Figure 6 Time-dependent inhibition of Mix-induced inflammation state in endothelial lysates and secretomes. A) PCA plots of lysate and secretome samples in conditions as indicated. Numbers indicate timepoints (4h, 8h and 24h). Lines show average trajectories per condition. Dotted line indicates samples at the same time point. Color indicates conditions, unstimulated (grey), Mix (red), Mix + JAKi (pink), Mix + IKK2/STAT3i (darkred). B) Significant hits of conditions as indicated versus unstimulated control per respective timepoint in lysates and secretomes.

Supplemental Figure 7

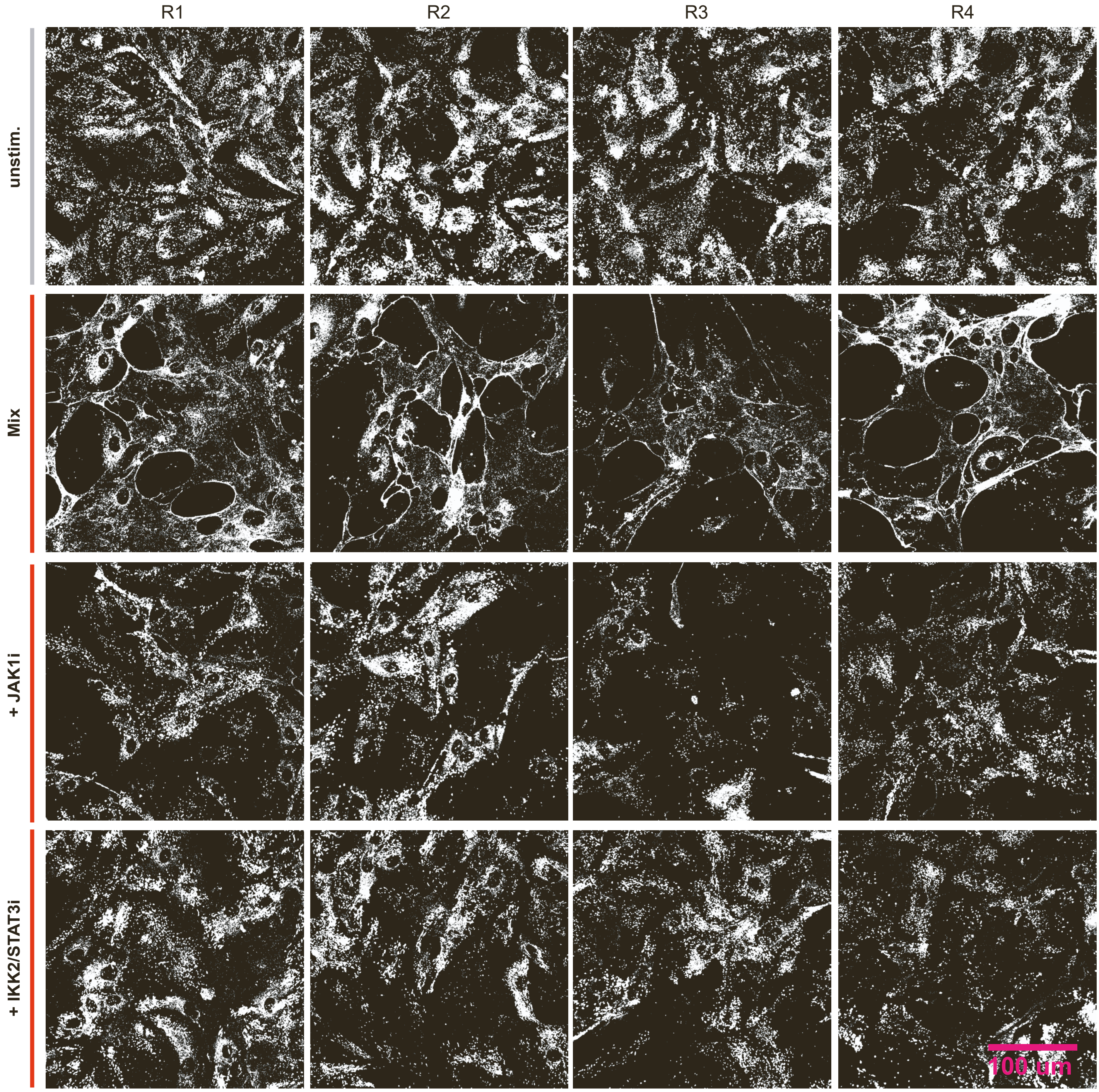

Supplemental Figure 7 Mix-induced VWF strings are not visible in combination with JAK1i or IKK2/STAT3i. Tresholded confocal images of ECs stained with anti-VWF (white). Scale bar (pink) indicates 100 µm. Treshold was set equally across images, representative image shown per replicate (N = 4 independent experiments, 3 images were analysed per experiment).

Supplemental Figure 8

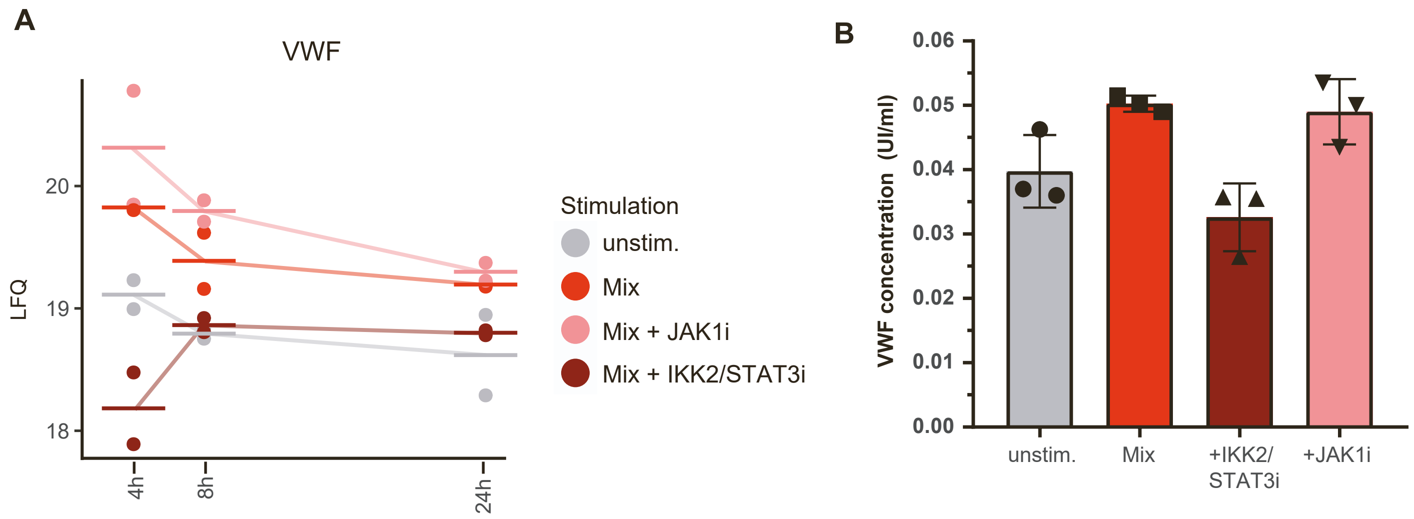

Supplemental Figure 8 Mix-induced secreted VWF levels are blocked by IKK2/STAT3i, but not JAK1i. A) LFQ levels of VWF in secretome at 4, 8 and 24h in conditions as indicated. Color indicates conditions, unstimulated (grey), Mix (red), Mix + JAKi (pink), Mix + IKK2/STAT3i (darkred). N =2 bioligical replicates, crossbar indicates mean. B) VWF levels in supernatant after 24h stimulation measured by ELISA in conditions as indicated. Data are represented as mean +/- SD (N = 3 biological replicates). No significant differences compared to unstimulated cells (p value > 0.05, Mann Whitney test)

Supplemental Figure 9

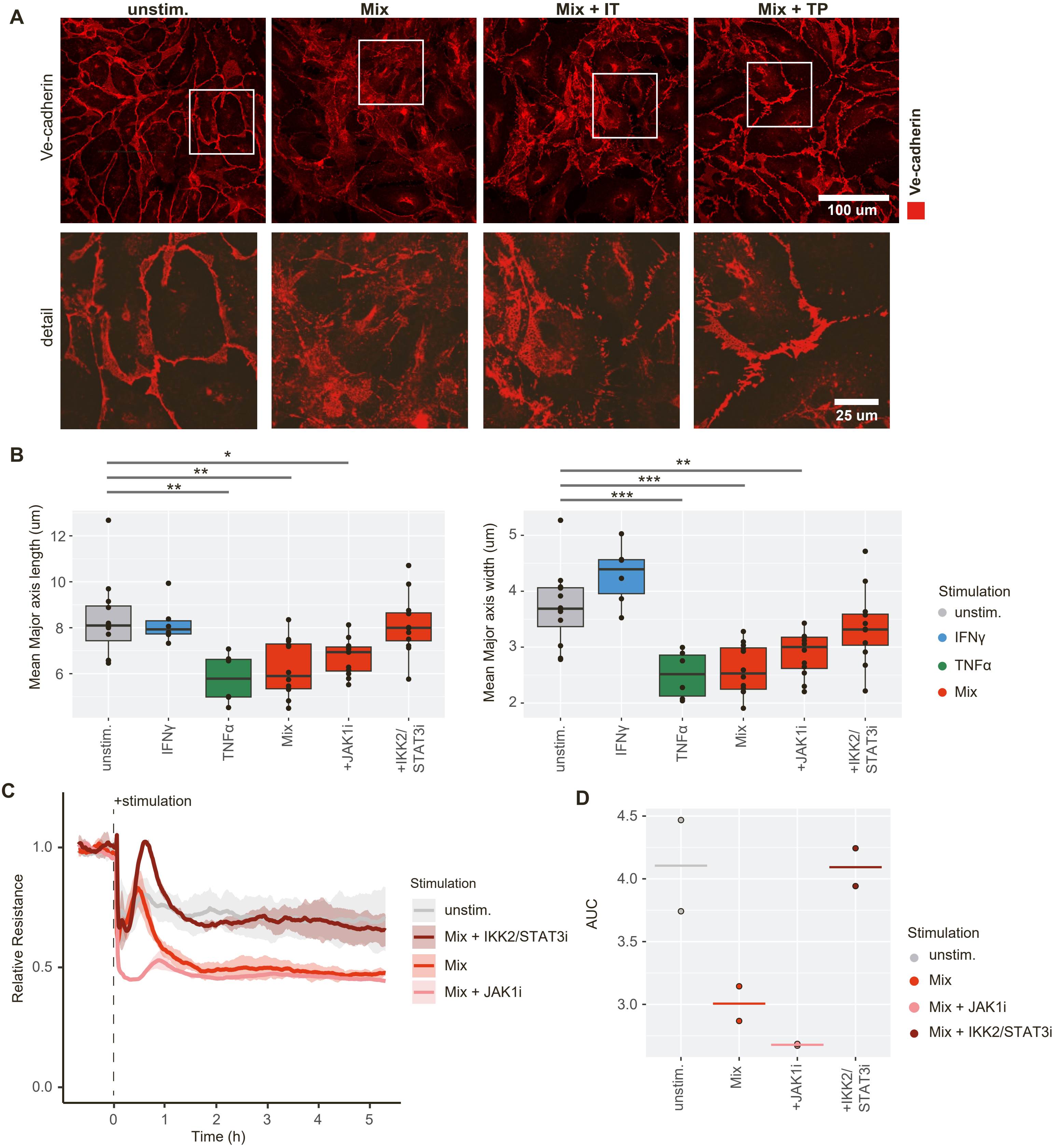

Supplemental Figure 9 Disruption of tight-junctions is blocked by IKK2/STAT3i, but not JAK1i. A) Confocal images of ECs stained with anti-Ve-cadherin (red) antibodies in conditions as indicated. Composites are shown in Figure 2D. Sale bar (white) indicates 100 μm for overall image and 25 μm for detail images, Brightness & contrast were adjusted equally across images, representative image shown (N = 4 independent experiments, 3 images per experiment). B) Boxplot of mean major axis object length and width (proxy for junction length and width respectively) per image. Middle line indicates median, upper and lower parts of boxplots indicate the 25th and 75th percentiles (N = 4 independent experiments for unstimulated and mix conditions, N = 2 independent experiments for TNFα and IFNγ stimulated conditions, 3 images were analysed per experiment). Color indicates conditions, unstimulated (grey), IFNg (blue), TNFa (green), Mix (red). Significant differences are indicated: * = adj. p-value < 0.05, ** = < 0.01, *** = < 0.001 (one-way-Anova and Tukey post-hoc test). C) Relative resistance levels over 6 h in conditions as indicated. Dotted line indicates addition of stimulus plus inhibitors, resistance levels were normalized to mean resistance over 30 minutes prior to addition of stimuli. Line represents mean across replicates, ribbon indicates standard deviation (N = 2 replicates). Color indicates conditions, unstimulated (grey), Mix (red), Mix + JAKi (pink), Mix + IKK2/STAT3i (darkred). D) Area under the curve of plot in C from addition of stimulus, crossbar indicates mean.

Supplemental Tables

Supplemental Table 1

	Inhibitors	Cat# (Selleckchem)	Target	Pathway	IC50	Final concs./ inhibitor (uM)
**1**	QNZ (EVP4593)	S4902	NFKB	NFKB-pathway	11 nM	0.1 1 10
**2**	TPCA-1	S2824	IKK2/STAT3	NFKB-pathway	17,9 nM	0.1 1 10
**3**	JSH-23	S7351	NFKB	NFKB-pathway	7.1 uM	1 10 100
**4**	SH-4-54	S7337	STAT3/STAT5	JAK/STAT pathway	300 nM; 464 nM	1 10 100
**5**	Itacitinib	S7812	JAK1	JAK/STAT pathway	2 nM	0.1 1 10
**6**	AS1517499	S8685	STAT6	JAK/STAT pathway	21 nM	0.1 1 10
**7**	Ritlecitinib	S8538	JAK3	JAK/STAT pathway	33,1 nM	0.1 1 10

Supplemental Table 1 Overview of used inhibitor panel. Category number, protein target, main pathway target, IC50 and concentrations used, are indicated per inhibitor.
